# Recycling of metals from LiFePO_4_ battery cathode material by using ionic liquid based-aqueous biphasic systems†

**DOI:** 10.1039/d4ra00655k

**Published:** 2024-03-19

**Authors:** Xiaohua Li, Maia Benstead, Nand Peeters, Koen Binnemans

**Affiliations:** a KU Leuven, Department of Chemistry Celestijnenlaan 200F, P. O. Box 2404 B-3001 Leuven Belgium Xiaohua.li@kuleuven.be; b Durham University, Department of Chemistry Durham DH1 3LE UK

## Abstract

Lithium-ion batteries are essential for electric vehicles and energy storage devices. With the increasing demand for their production and the concomitant surge in waste generation, the need for an efficient and environmentally friendly recycling process has become imperative. This work presents a new approach for recycling of metals from the LiFePO_4_ (LFP) cathode material. The cathode material was first leached by a HCl solution without an oxidizing agent. Subsequently, an ionic-liquid-based aqueous biphasic system (IL-based ABS) was used for the separation of lithium and iron from leachate solutions, followed by a precipitation process. The influence of the acid concentration, solid-to-liquid ratio and leaching time on the leaching yield was investigated. UV-vis absorption spectra revealed the presence of mixed-valent iron in the leachate, with 83 ± 1% Fe(ii) and 17 ± 1% Fe(iii). The ABS systems comprised tributyltetradecylphosphonium chloride [P_44414_]Cl and a salting-out agent (HCl or NaCl). The extraction percentage of iron reached 90% and less than 1% of lithium was extracted under the studied optimal conditions. Further enhancement of iron extraction, reaching 98%, was achieved *via* a two-stage cross-current extraction process. Iron was precipitated from the loaded IL phase with an efficiency of 97% as Fe(OH)_2_ and Fe(OH)_3_, using an aqueous ammonia solution. Lithium was precipitated as Li_3_PO_4_ with a lithium purity of 99.5% by adding K_3_PO_4_ solution. The ionic liquid used in the process was efficiently regenerated and used in four extraction cycles with no activity decline, with an extraction percentage of 90% of iron in each cycle.

## Abstract 2

Lithium and iron were recycled from LiFePO_4_ battery cathode material *via* leaching, separation by ionic liquid-based ABS and precipitation.

## Graphical abstract

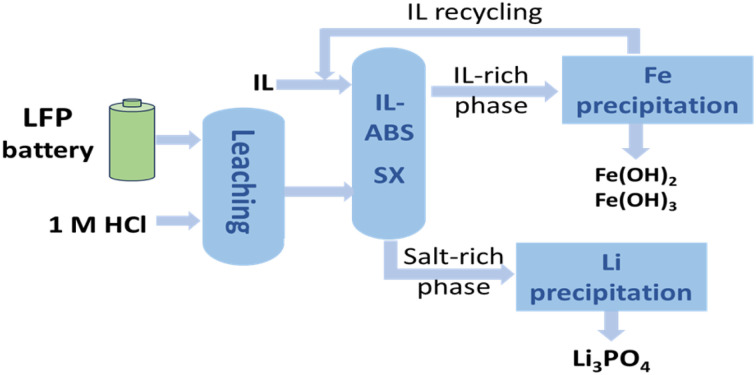

## Introduction

Lithium-ion batteries (LIBs) have been the primary source for electrical energy storage and are used in many electrical devices and electric vehicles.^1–3^ The end-of-life LIBs need to be treated properly, as sending spent LIBs to landfill is detrimental to the environment and also unacceptable in the circular economy.^4,5^ The cheapest LIBs for (hybrid) electric vehicles and energy storage devices use lithium iron phosphate LiFePO_4_ (LFP) as the cathode active material.^2,6,7^ Although LFP batteries do not contain valuable metals such as nickel and cobalt, recycling of these batteries is still relevant due to their lithium content.^7^

LFP recycling is typically done by a hydrometallurgy process, where metals are leached by an acidic solution, following by separation of the metals *via* precipitation or solvent extraction.^2,8,9^ Li and co-authors used a H_2_SO_4_/H_2_O_2_ system to selectively leach lithium from LFP material, and Fe(ii) oxidized by H_2_O_2_ to Fe(iii), which reacts with PO_4_^3−^ to form a FePO_4_ precipitate.^8^ Other similar systems employing an acid and an oxidizing agent were also investigated, such as H_2_SO_4_/Na_2_S_2_O_8_, HCl/NaOCl, HCl/H_2_O_2_ and organic acid/H_2_O_2_.^9–13^ In these systems, lithium was selectively leached to the solution and Fe(ii) was oxidized to Fe(iii), with simultaneous formation of FePO_4_ precipitate. Precipitation as a method to recover Fe and Li from spent LFP batteries is a relatively simple process, but this method has drawbacks. Coprecipitation of lithium and other metal impurities present in the battery can occur. The FePO_4_ precipitates after leaching are present in the solid residue, making the reuse of FePO_4_ difficult. Therefore, it is preferable to first leach both Li and Fe into solution, followed by their separation *via* solvent extraction.

Conventional solvent extraction system consists of an organic phase and an aqueous phase. One alternative extraction system is aqueous biphasic system (ABS), where water is the solvent of both phases.^14,15^ A conventional ABS is composed of two immiscible aqueous phases with each phase rich in one solute, based on polymer–polymer, polymer–salt and salt–salt combinations.^16,17^ Ionic liquid based ABS (IL-based ABS) is composed of an IL and a second solute, such as inorganic or organic salts, polymers or amino acids.^18–22^ In recent decade, IL-based ABS has been effectively applied in the separation of metal ions.^23–25^ By employing IL-based ABS, we can maintain the high selectivity of ILs towards metal ions. Moreover, the high viscosity of the IL – one of the main barriers for the industrialization of IL applications, could be mitigated by using the IL-based ABS, as the IL-rich phase usually has lower viscosity than the pure IL.^26^

In this work, we studied recycling of metals from the cathode material of LiFePO_4_ battery, by first leaching lithium and iron by hydrochloric acid without any oxidizing agent, then separating them by solvent extraction using an ionic-liquid-based aqueous biphasic systems (IL-based ABS). The employed IL was tributyltetradecylphosphonium chloride ([P_44414_]Cl) that is the most popular IL to form ABS for extraction of metals, because of the strong complexing ability of chloride ions with metals.^24,27,28^ Metals were recovered from both the extractant and the raffinate phase to enable the reuse of the IL system.

## Experimental

### Materials

The LFP cathode material (LiFePO_4_) was obtained from Vito NV (Mol, Belgium) who bought it from Xiamen Tob New Energy Technology Co., Ltd. (Xiamen, China). The ionic liquid tributyltetradecylphosphonium chloride, [P_44414_]Cl, (98%) was ordered from ABCR (Karlsruhe, Germany). HNO_3_ (65%) and H_2_O_2_ (30 wt%) were purchased from Chem Lab (Zedelgem, Belgium). HCl (37%), NaCl (>99.5%) and NaOH (pearls) were from Fisher Chemical (Belgium). Ethylene glycol (99.5%) was from Thermo Fisher Scientific (Geel, Belgium). Iron(ii) chloride (98%), 1,10-phenanthroline (>99%), potassium antimony tartrate (>99%), ammonia solution (25%) and ascorbic acid (>99%) were obtained from Sigma Aldrich (Diegem, Belgium). Ammonium molybdate (>99%) was ordered from Acros Organics (Geel, Belgium). Hydranal Composite 5, Hydranal Methanol dry and hydroxylamine (98%) were from Honeywell Fluka (Seelze, Germany). The ICP standard solutions (Li, Fe, V, Al, Si, Cs, Sc, P, Na, Mn) were ordered from Chem Lab (Zedelgem, Belgium). All materials were used as received, without any further purification.

### Instrumentation

The concentration of metal ions present in a solution was determined by inductively coupled plasma optical emission spectroscopy (ICP-OES) analysis with a PerkinElmer Avio 500 ICP-OES spectrometer. Both the aqueous solutions and the IL samples were diluted in 5 wt% HCl solution with addition of scandium as an internal standard and cesium as an ionization buffer for lithium analysis.

A Bruker S2 Picofox total reflection X-ray fluorescence (TXRF) spectrometer with Mo source, operating at 50 kV and 600 μA was used to qualitatively determine the element concentrations in the solid materials. Samples were prepared by suspending approx. 3 mg of the solid in a 50 vol% solution of Triton X-100, in ultrapure water. A 3 μL aliquot was pipetted onto a quartz carrier, which was pre-treated with 30 μL of Serva silicone solution in isopropanol (to prevent spreading of the droplet, dried for 20 min at 60 °C). The samples were dried in an oven at 60 °C for an hour prior to measuring. The measurement time was 300 s per sample.

A Speedwave Xpert microwave digester (Berghof, Germany) was used to digest the solid material into HNO_3_ acid solution under high pressure and temperature. A Bruker D2 phaser with Cu Kα radiation at 30 kV and 10 mA in the measurement range 2*θ* of 5–120° was used for X-ray diffraction (XRD) measurements of the LFP powder and the solid residue after leaching. Data processing was performed with the X'Pert HighScore software. An Agilent Cary 6000i UV-vis-NIR spectrophotometer was used for measuring the UV-vis spectra from 200 nm to 1000 nm. All measurements were conducted in quartz glass cuvettes with a 10 mm pathlength. A Mettler-Toledo V30S volumetric Karl-Fischer titrator was used for the measurements of water content in the IL-rich phase. To determine the viscosity and density of the loaded IL phase, a Lovis 2000ME/DMA 4500 M, viscosity/density meter was used.

### LiFePO_4_ cathode material characterization

The mineralogical composition of the LFP cathode material powder was characterized by the XRD analysis (analytical details in the Instrumentation section). Furthermore, the solid material was directly analyzed by TXRF after suspending in solutions, to determine the purity and composition of the cathode material. In addition, the solid material was completely digested into 65% HNO_3_ solution with a microwave digestion instrument. Concentration of elements in the obtained solution was determined by ICP-OES analysis after dilution.

### Leaching of LiFePO_4_

Leaching of the LFP powder was carried out in 10 mL glass vials at room temperature. Several variables were studied to obtain the optimal conditions, including hydrochloric acid concentration (0–5 M), solid-to-liquid ratio (10–100 g L^−1^) and leaching time (0.5–6 hours). Metal concentration in each leachate solution was determined by ICP-OES analysis. Leaching efficiency was calculated using eqn (1), where *m*^leachate^_i_ is the mass of metal i in the leachate and *m*^solid^_i_ is the total mass of metal i in the solid LiFePO_4_ battery material:1
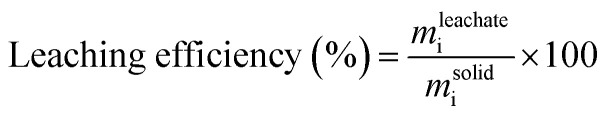


After leaching of the LiFePO_4_ material, a black solid residue remained and was collected. This was dried in an oven overnight to remove the excess of water. The solid was measured using XRD after grinding into a fine powder using a pestle and mortar. Some of the remaining solid (5 mg) was digested using 10 mL of a 65% HNO_3_ with the same microwave digestion instrument as the one for the LFP material. The digested samples were then measured using ICP-OES for elemental concentration determination.

The oxidation state of the iron ions in the leachate solution were determined by UV-vis absorption spectroscopy. The samples were prepared following a similar procedure as the reported literature.^29^ Shortly, 0.1 mL of leachate solution, 1 mL of sodium acetate buffer solution, and 1 mL of 1,10-phenanthroline (*o*-phen) solution (0.1 wt%) were added to ethylene glycol to make up to a 10 mL solution. The standard solutions with 2–10 ppm Fe(ii) were prepared using FeCl_2_ in ethylene glycol together with the buffer and 1,10-phenanthroline solution.

### Solvent extraction with ABS system

Separation of metals from the leachate solutions were carried out with a [P_44414_]Cl-based ABS. Several variables were investigated for the ABS extraction systems, including the IL concentration, the types of the salting-out agent and the temperatures. For every extraction system, 3 mL of aqueous leachate solution (with designed concentration of HCl or NaCl) and 2 mL of IL solution (with 10–90 wt% of IL in water) were mixed in a 15 mL centrifuge tube. The sample was shaken for 1 hour at 200 rpm. For the experiments conducted at room temperature, the mixture was then centrifuged for 5 min at 4000 rpm to facilitate phase separation. For experiments conducted at 50 °C, the samples were put in a water bath for 4 hours for equilibration. The volume of each phase was recorded after phase separation. The metal concentrations in each phase were determined by ICP-OES analysis after dilution.

The distribution ratio (*D*) and the extraction percentage (*E*%) of the metal was calculated *via*eqn (2) and (3):2
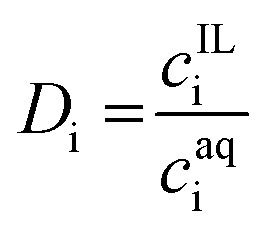
3
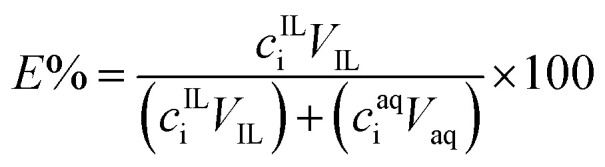
where *c*^IL^_i_ and *c*^aq^_i_ are the concentrations of the metal i in the IL and the aqueous phases, respectively. *V*_IL_ and *V*_aq_ are the volumes of the IL and the aqueous phases after phase separation, respectively.

### Precipitation stripping of iron

Precipitation stripping was employed for the removal of iron from the IL phases. Aqueous ammonia (25 wt%) solution was added to the isolated IL phase in excess, in a 3 : 1 molar ratio of iron to ammonia. The samples were shaken for 30 min at 200 rpm, followed by centrifuging for 1 hour at 5000 rpm to facilitate the solid and liquid separation. The concentrations of iron in the IL before and after precipitation (*c*^IL before^_Fe_ and *c*^IL after^_Fe_) were determined by ICP-OES analysis, which was used for calculation of the percentage precipitation of iron (eqn (4)):4
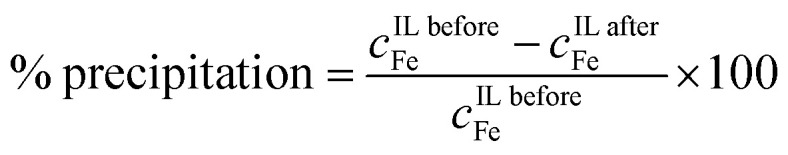


### Recycling of P_44414_Cl

IL was regenerated for recycling after precipitation stripping of iron and evaporating the excess ammonia and water on a vacuum rotary evaporator. The regenerated IL solution (90 wt%) was then contacted with fresh leachate solution with 4.5 M NaCl for separation of metals at a volume ratio of 3 : 2 (aqueous : IL). The mixture was shaken at 200 rpm for 60 min at room temperature, followed by centrifuging for phase separation. The procedures were repeated 3 times to reuse the IL for 4 cycles. The experiments were performed in triplicate.

### Precipitation of lithium

A raffinate obtained after 2-stage cross-current solvent extraction steps using 4.5 M NaCl in the leachate and 90 wt% IL solution at a volume ratio of 3 : 2 was used for the precipitation test of lithium. Each raffinate (3 mL) was first neutralized by NaOH (5 M) solution to the targeted pH (5–9), which was afterwards shaken for 60 min at room temperature to allow the precipitation of iron, followed by filtration. Then 80 μL of K_3_PO_4_ (2 M) solution was added to each filtrate solution (2 mL) for lithium precipitation. The filtrate solutions after adding NaOH and those after adding K_3_PO_4_ were analyzed by ICP-OES after dilution, to determine the metal concentrations.

## Results and discussion

### Characterization of the LFP cathode material

XRD analysis on the LFP powder showed that the material is pure LiFePO_4_ phase (see Fig. S1 in the ESI†). TXRF analysis on the solid material after suspending in a solution confirmed the presence of Fe and P, as well as trace amounts of Ca, Cr, Mn, Ni and V (Fig. S2†). The concentration of these elements was then determined through ICP-OES analysis of the microwave digested solid. Besides the main elements Li, Fe and P, only vanadium was detectable *via* ICP-OES analysis (Table 1). The concentrations of Li and Fe in the LFP solid are very close to those of the theoretical value for the pure LiFePO_4_ compound, again confirming that the cathode material was mainly LiFePO_4_ with little impurities. For simplification, the rest of the impurities found by TXRF were ignored as they were untraceable by ICP-OES, so only recycling of Li and Fe from the LFP material was investigated further.

**Table 1:** Elemental composition of the LFP material and the theoretical values in pure LiFePO_4_ compound

Element	Content in LFP solid (wt%)	Theoretical composition of LiFePO_4_ (wt%)
Li	4.3 ± 0.1	4.4
Fe	38.1 ± 0.2	35.4
V	0.3	0.0
Others	57.3	60.2

### Leaching of metals from the LFP cathode material

Prior to separation of Li and Fe, the metals were first leached from the cathode material using HCl solution. No oxidizing agent was added to prevent the precipitation of FePO_4_. HCl was selected rather than other mineral acids, in order to avoid the anion exchange in the following solvent extraction processes where the chloride IL was employed. The leaching conditions were optimized in terms of the acid concentration, solid-to-liquid ratio and leaching time.

**Fig. 1:**
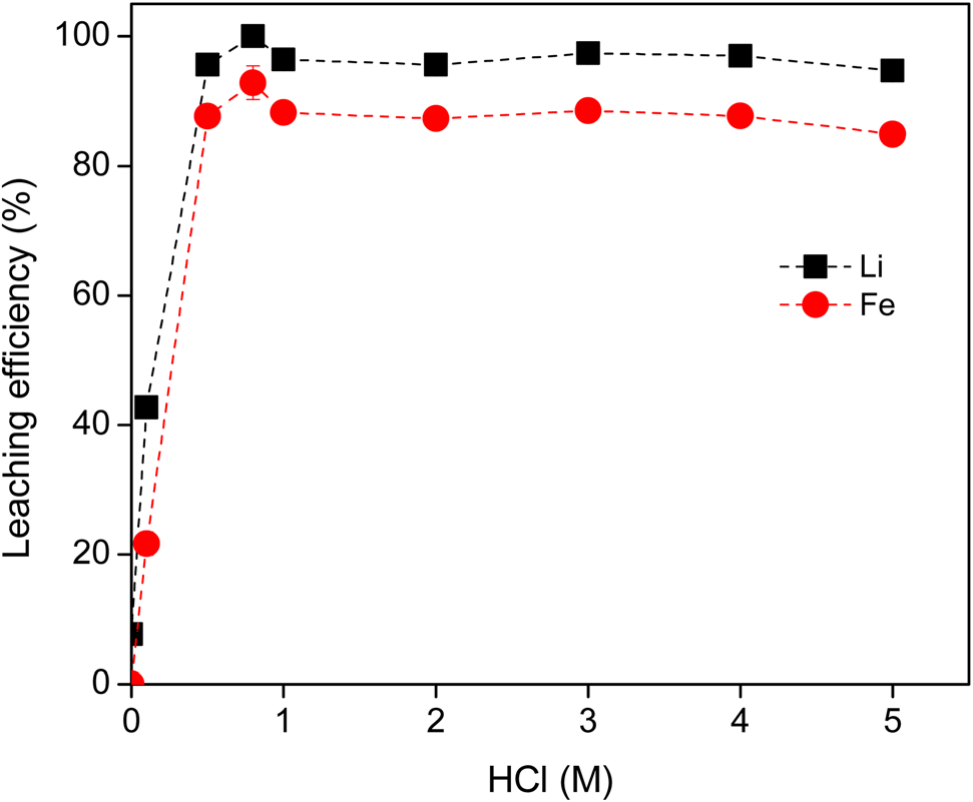
Effect of acid concentration on the leaching efficiency of Li and Fe from LFP material, S/L = 20 g L^−1^, 2 hours, 500 rpm and room temperature. The invisible error bars indicate very small errors.

The HCl concentration was varied from 0 to 5 M, at a solid-to-liquid ratio of 20 g L^−1^ and the leaching experiments were carried out at room temperature. The leaching efficiencies of both Li and Fe did sharply increase when the acid concentration was increased from 0 M to 1 M, reaching a maximum of 97% for Li at 1 M HCl (Fig. 1). The leaching efficiency of Li and Fe remained approximately constant above 1 M. There was low selectivity in the leaching of the LFP battery material by HCl. All ions were leached into solution to similar percentages. Higher acid concentrations did not greatly increase the concentration of Li and Fe ions in solution.

**Fig. 2:**
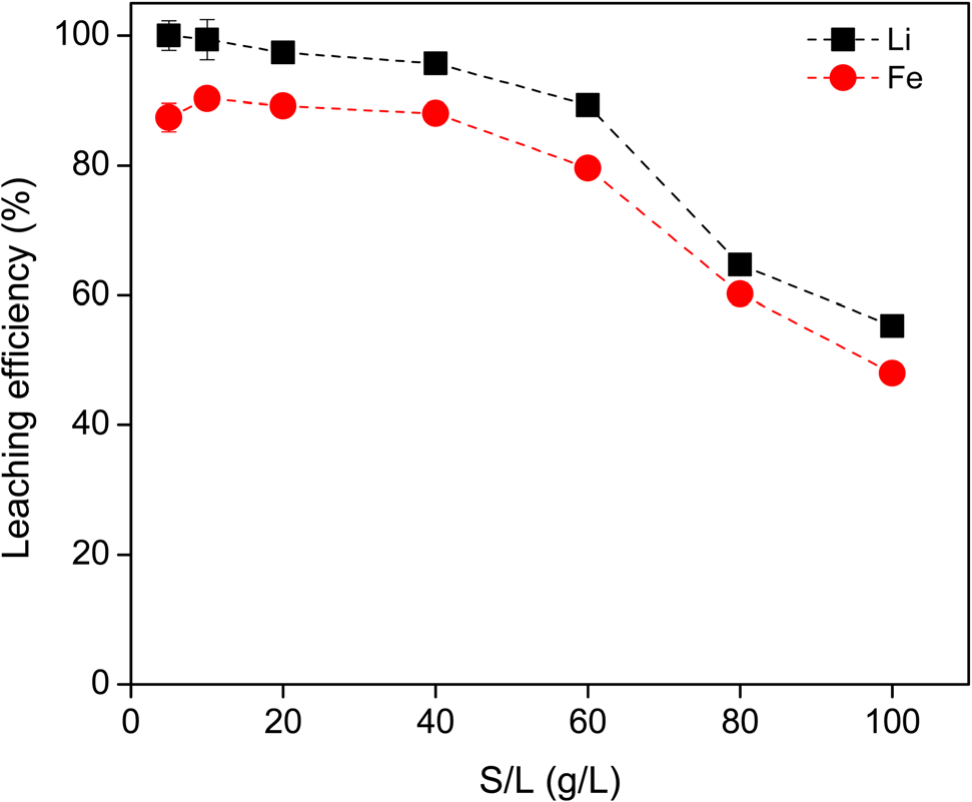
Effect of solid to liquid ratio on the leaching efficiency of Li and Fe using 0.8 M HCl, 3 hours, 500 rpm, room temperature. The invisible error bars indicate very small errors.

The S/L ratio was further investigated using 0.8 M HCl for the leaching of Li and Fe (Fig. 2). As the S/L ratio increased from 5 to 40 g L^−1^, the leaching efficiency of Li remained above 95%, while that of Fe remained almost constantly above 85%. At 60 g L^−1^ and higher, the leaching efficiency for both metals were significantly reduced. The solution became saturated with solid, so the diffusion and dissolution of the metal ions was hindered. This resulted in a lower leaching efficiency. Therefore, the S/L ratio of 40 g L^−1^ was chosen as optimal for economic reasons.

**Fig. 3:**
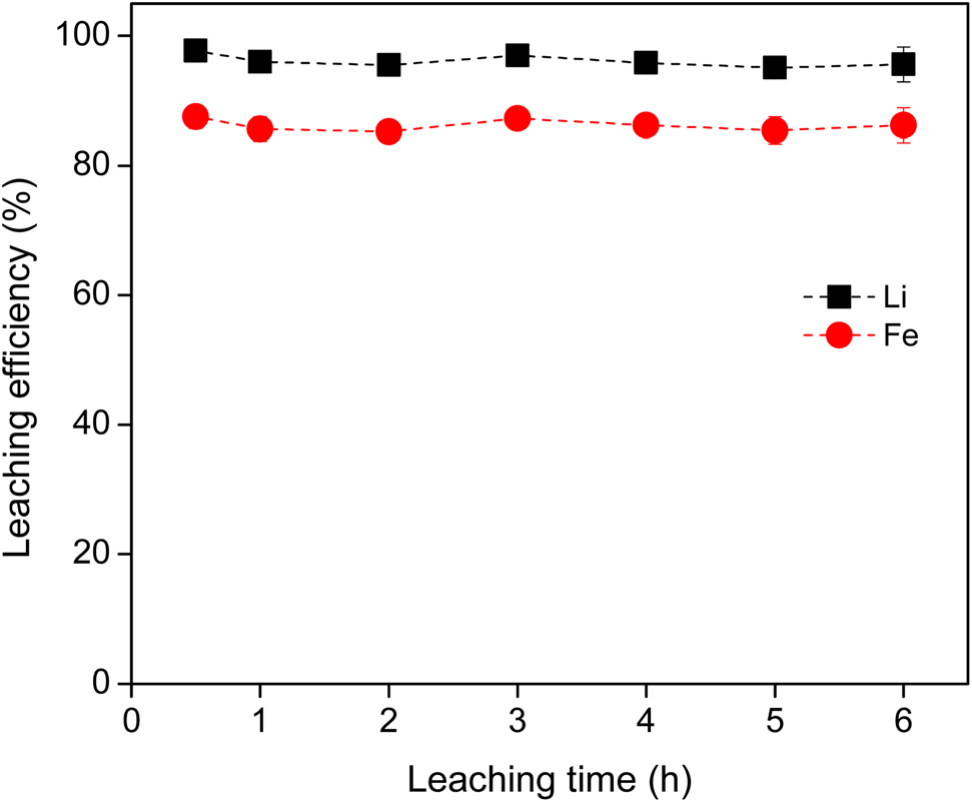
Relationship between leaching time and leaching efficiency (%) of Li and Fe from LFP material, using 0.8 M HCl, with a S/L ratio of 10 and 40 g L^−1^, at room temperature. The invisible error bars indicate very small errors.

The leaching rate of metals from LFP material was determined using 0.8 M HCl at a S/L ratio of 40 g L^−1^ at room temperature (Fig. 3). The leaching efficiency for Li and Fe reached maximum within 30 min and kept constant thereafter, indicating that leaching was very fast under the studied conditions. Moreover, no selectivity was observed in terms of time, *i.e.*, leaching of Li ad Fe occurred simultaneously through the leaching process. For a large scale, slightly longer leaching time might be needed. Therefore, from the above experimental results, the optimum leaching conditions of LFP cathode material were found to be: (0.8–1.0) M HCl, 40 g L^−1^, 2 hours, 500 rpm and room temperature.

### Characterization of the leaching residue

After leaching, a solid black residue remained. The solid residue was digested using 65% HNO_3_ and analyzed by ICP-OES. The residue was found (by mass) to contain trace amounts of Li (0.3 ± 0.1 wt%) and Fe (4.7 ± 0.4 wt%) which remained due to incomplete leaching and Al (0.3 wt%) and Si (0.5 wt%). The Al and Si could have been present in the original LFP sample but in such low quantities that they could not be detected by ICP-OES analysis after digestion of the LFP powder. After leaching, the mass of solid was reduced, increasing the concentration of Al and Si present in the sample, thus they could then be detected using the same ICP dilution procedure as for the LFP solid.

The solid was investigated by XRD analysis. The diffraction pattern can be seen in Fig. S3.† Large amounts of background noise were present, and no discernible, sharp peaks were formed. This indicates that the analyzed material was largely amorphous. As described on the company website, the purchased LFP material contains 1.29% of carbon. Therefore, the black residue could be graphite.

### Leaching mechanism

The leaching mechanism was investigated by studying the oxidation states of the iron ions in the leachate. Knowing whether iron is present in the divalent or trivalent form is important, because the extraction behavior of Fe(iii) and Fe(ii) is usually much different. It is known that iron in LFP is in its divalent form. In most studies, an oxidant such as H_2_O_2_ was used for leaching of LFP material in an acid solution, so that Fe(ii) was oxidized to Fe(iii) during the leaching process.^9,11^ In our study, no oxidizing agent was added to the HCl solution, the iron ions in leachate were mostly at the same oxidation state as that in the LFP, *i.e.*, as Fe(ii). This was confirmed by the UV-vis absorption spectra of the leachate, by using a colorimetric agent, 1,10-phenanthroline, which can form a colored complex with the Fe(ii) ion.

The concentrations of Fe(ii) and Fe(iii) in the leachate were further quantified by UV-vis spectrophotometric analysis. For the leachate samples, two solutions were prepared, one with the original leachate and one with hydroxylamine added. Hydroxylamine reduces all Fe(iii) in the sample to Fe(ii).^30^ The difference in the measured concentrations of Fe(ii) in the two solutions was then used to determine the concentration of Fe(iii) in the leachate. The results of the UV-vis analysis are present in the ESI. It was found that the leachate contained approximately 83 ± 1% Fe(ii) and 17 ± 1% Fe(iii). The presence of Fe(iii) could be attributed to the oxidation of Fe(ii) to Fe(iii) by the oxygen in the atmosphere.

### Separation of metals using an IL-based ABS solvent extraction system

The separation of Li and Fe from the leachate was investigated in IL-based ABSs, as these two metals were the main components present in the leachate. The other metals that were present in trace amounts were neglected. The ionic liquid tributyltetradecylphosphonium chloride, [P_44414_]Cl, was selected, because it has been employed in various ABS systems for extraction of metals.^24,27,28,31^ Two salting-out agents selected in this study were HCl and NaCl.

### [P_44414_]Cl–HCl ABS

To avoid introducing new type of ions to the system, HCl is preferred over NaCl, since acid could also act as the salting-out agent to induce the formation of ABS, though HCl influences the speciation of the phosphate group in the solution.^32,33^ Therefore, the ABS based on [P_44414_]Cl and HCl were first studied for the separation of Li and Fe. [P_44414_]Cl-based ABS exhibits a lower critical solution temperature (LCST), thus two phases preferentially form and the system demixes, as the temperature is raised.^33^ Using a slightly higher temperature is preferential over a higher acid concentration for the HCl system. A higher acid concentration would impact the downstream processing when recycling the ionic liquid and separating the metals. Therefore, to facilitate the phase separation and to allow lower concentrations of HCl, a temperature of 50 °C was employed in the [P_44414_]Cl–HCl-based ABS.

**Fig. 4:**
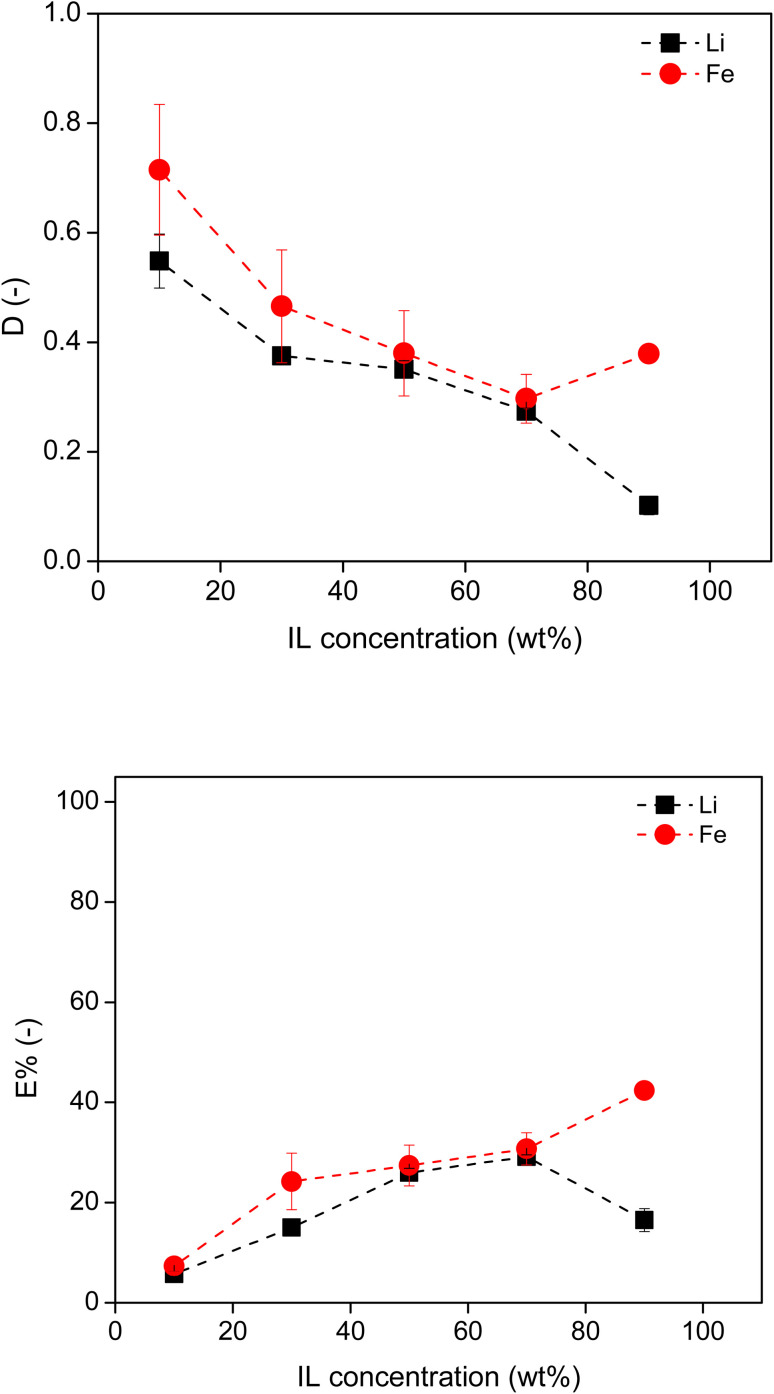
Distribution ratio and extraction percentage of Li and Fe in [P_44414_]Cl–HCl system, using 2.5 M HCl and varying wt% of IL, in a 3 : 2 aqueous : IL volume ratio, at 50 °C.

The effect of the [P_44414_]Cl concentration on the extraction of metals was studied by varying the IL concentration from 10 wt% to 90 wt% in the original IL solution. This IL solution was mixed with the leachate containing 2.5 M HCl at a volume ratio of 2 : 3 (IL : leachate) at 50 °C for 4 hours to attain equilibrium. The *D* and *E*% of Li and Fe in terms of IL concentration are shown in Fig. 4. As the IL concentration increased, the *D*_Li_ and *D*_Fe_ values became smaller and the *E*% of both metals increased in most of the tested systems. This is attributed to the increasing volume of the IL phase, which increased the absolute amount of metal ions, but reduced the concentration of metals in the IL phase, leading to a larger *E*% and a lower *D*. However, among all the tested systems, both *D*_Li_ and *D*_Fe_ were less than 0.7 and the *E*% of both metals were less than 42%. Moreover, there was little extraction selectivity towards Fe. This suggests that HCl is a poor salting-out agent, resulting in large volumes of water within the IL phase, and thus poor extraction efficiency. The low extraction efficiency of Fe could be attributed to two factors: (1) the relatively low acid concentration and (2) the fact that the majority of the iron ions were in the Fe(ii) rather than the Fe(iii) state. Here 2.5 M HCl was employed in the leachate solution, which would be 1.5 M HCl regarding the whole ABS system. This concentration is much lower than the value (8 M HCl) reported for effective extraction of Co using an acid-based ABS.^25^

**Fig. 5:**
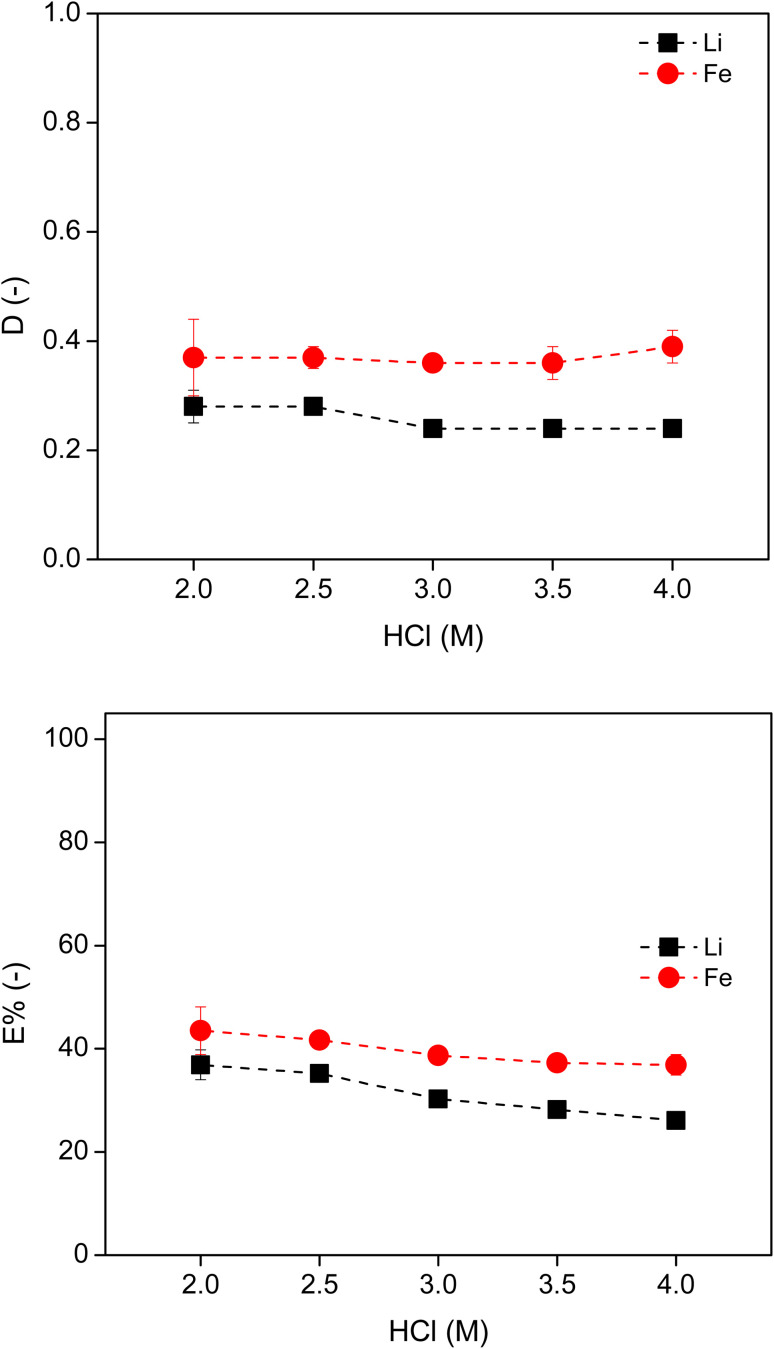
Distribution ratio and *E*% of Li and Fe in the [P_44414_]Cl–HCl system, using 90 wt% IL and varying HCl concentrations, in a 3 : 2 aqueous : IL volume ratio, at 50 °C. The invisible error bars indicate very small errors.

Next, the concentration of HCl was varied from 2 M to 4 M using 90 wt% [P_44414_]Cl for the extraction of Li and Fe at 50 °C. A higher HCl concentration was not selected to limit the consumption of base in the following precipitation stripping of lithium. As the concentration of HCl increased, both the *D* and *E*% of each metal remained fairly constant (Fig. 5). This was due to the large volume of free water in the system which was able to solvate Li^+^ in the IL phase. It was found that nearly 50 vol% of water was in the IL-rich phase. Furthermore, HCl can also be co-extracted by the IL.^34^ The volume of IL-rich phase showed a slight decrease, *e.g.*, from 3.4 mL to 3.0 mL as the HCl concentration increased from 2 M to 4 M. This again confirms that HCl is a poor salting-out agent to induce the ABS. The use of HCl as a salting-out agent in this system was ineffective due to the low selectivity and low extraction percentage of Fe.

### [P_44414_]Cl–NaCl ABS

A stronger salting-out agent NaCl was further employed for the formation of ABS and for the separation of Li and Fe, by varying the concentration of IL and NaCl at room temperature. The IL concentration was varied from 10 to 90 wt% for the extraction of Li and Fe using 2 M NaCl in the original leachate (already containing 1 M HCl). However, of the five IL concentrations tested, only the systems with 70 wt% IL and 90 wt% IL formed two phases at room temperature. When the concentration of IL was lower than 70 wt%, sufficient water molecules were present to dissolve the IL so that the system was fully miscible. The *D* and the *E*% of Li and Fe in these two systems can be found in Fig. S7 in the ESI.† The extraction of Fe was lower than anticipated, reaching a maximum of 31% with 90 wt% IL, due to the insufficient salting-out agent and the nature of Fe(ii) that is difficult to be extracted in this system.

**Fig. 6:**
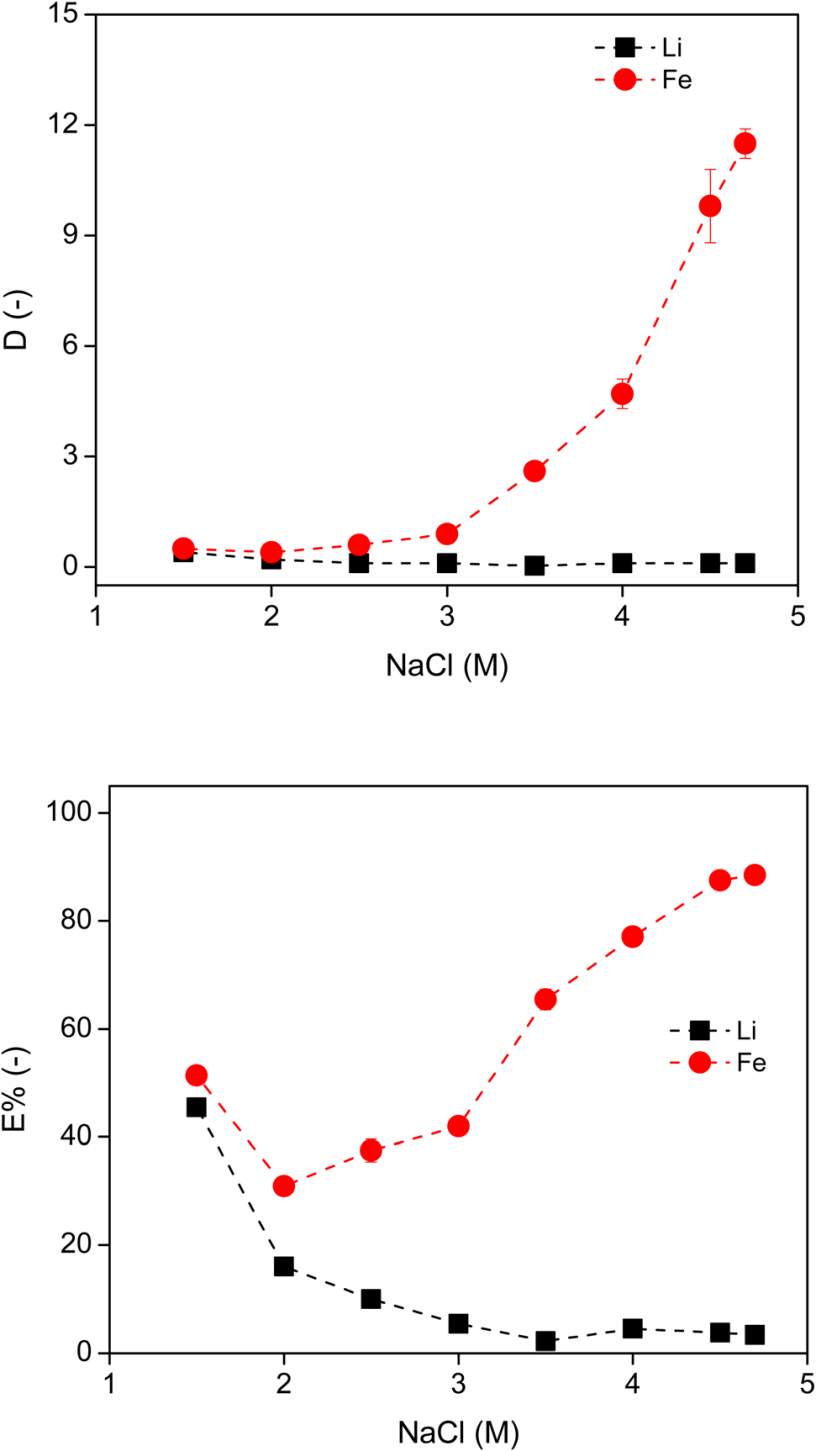
Distribution ratio and extraction percentage of Li and Fe in the [P_44414_]Cl–NaCl system, using 90 wt% IL in terms of the NaCl concentration, in a 3 : 2 aqueous : IL volume ratio at room temperature. The invisible error bars indicate very small errors.

Next, the concentration of NaCl in the leachate was varied from 1.5 M to 4.7 M (which represents the solubility limit) for the extraction of Fe and Li with 90 wt% IL. As the concentration of NaCl in the system increased, the *D*_Fe_ increased and *D*_Li_ reduced (Fig. 6). The *E*% of Li and Fe followed a similar trend. Increasing the amount of salting-out agent in the system reduced the amount of free water available to dissolve the IL, facilitating phase separation. The water molecules preferentially solvate the Na^+^ and Li^+^ cations, so that they remain in the aqueous phase, leading to a more hydrophobic IL phase. The initial high extraction percentage value of Li^+^ at 1.5 M NaCl was again due to the large amount of free water in the IL phase. At the solubility limit of NaCl (4.7 M) in the leachate, the maxima of *D*_Fe_ (=12) and *E*%_Fe_ (=88%) were achieved. These values are much lower than the reported data for extraction of Fe(iii) by a similar ABS system, where the *D*_Fe(iii)_ is higher than 10 000 and *E*% of Fe(iii) reaches 100%.^35^ Therefore, extraction of Fe(ii) is more difficult than that of Fe(iii) using this ABS.

**Fig. 7:**
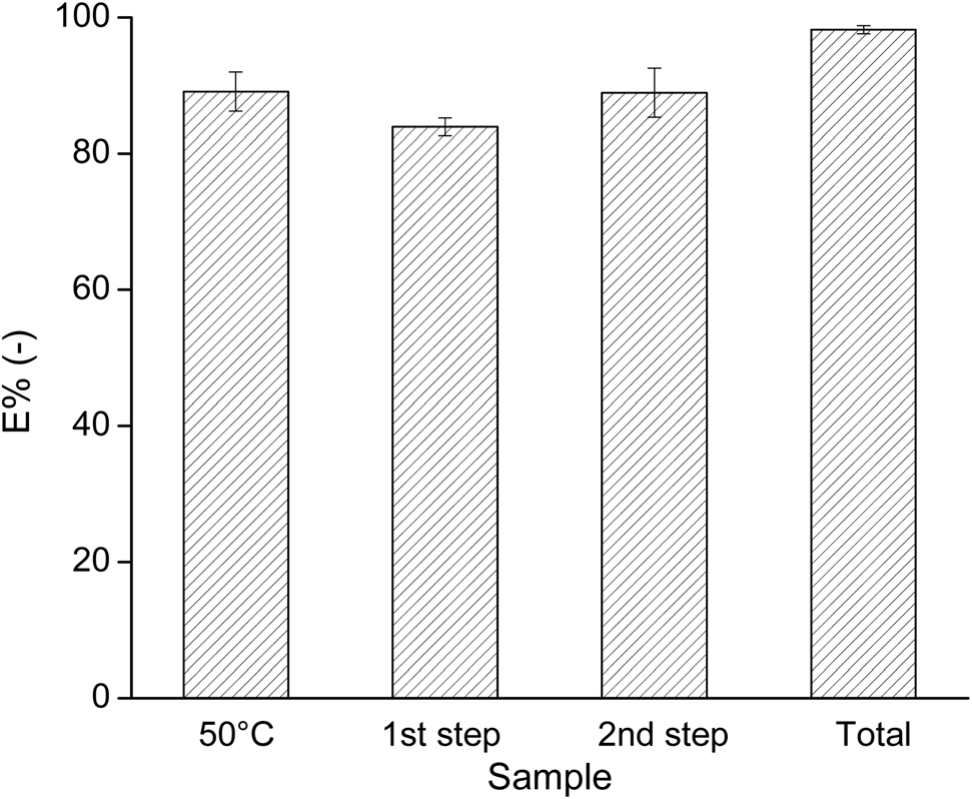
Extraction percentage of Fe from the aqueous phase by [P_44414_]Cl, using 90 wt% IL and a leachate with 4.5 M NaCl in a 3 : 2 aqueous : IL volume ratio, at 50 °C (the first column on the left) and at room temperature using two extraction steps (the remaining 3 columns).

To improve the extraction efficiency of Fe(ii) by IL, the effect of raising the temperature of the system was investigated. It is known that for a LCST system, an increase in temperature could enhance the phase separation. Thus, whether a better phase separation could induce a higher extraction efficiency of metals was tested by conducting the extraction experiment at 50 °C using the leachate with 4.5 M NaCl and IL solutions with 90 wt% [P_44414_]Cl, in a 3 : 2 aqueous : IL volume ratio. The *E*% of Fe by this system is shown as the first column on the left in Fig. 7. The system at 50 °C gave the same extraction percentage of Fe as the one at room temperature, *i.e.* about 90%. Hence, a higher temperature did not facilitate Fe extraction.

Next, two-stage cross-current solvent extractions were applied to increase the *E*% of Fe from the leachate solution with 4.5 M NaCl using 90 wt% IL in a 3 : 2 volume ratio. The *E*% of Fe in each extraction step and in total are presented by the 3 columns on the right of Fig. 7. For all tested systems, the coextraction of Li was 0% (within the error limits of the measurement), so that all the Li^+^ ions remained in the aqueous phase. Use of two extraction steps allowed quasi-quantitative Fe extraction (approx. 98 ± 1%).

As we have assumed the viscosity of the IL phase in solvent extraction processes could be reduced by using ABS compared to using pure IL, the viscosity of the IL-rich phase together with the water content was determined for the IL phase obtained in the 1st and 2nd extraction steps (Table 2). The viscosities of the IL phases in both extraction steps are much lower compared to those of the [P_666,14_]Cl system. Pure [P_666,14_]Cl has a viscosity of 1824 mPa s at 25 °C.^36^ After loading of divalent metal ions such as Co(ii) in the IL phase, the viscosity increased even more.^37^ The IL-rich phase in the ABS has indeed a lower viscosity than the conventional IL system. The viscosity of the IL is affected by factors such as water content, metal content and the charge of the extracted metal complexes. The IL-phase in the 2nd extraction step has a lower viscosity due to the lower metal loading, since most of the Fe(ii) ions have been extracted to the IL phase in the 1st extraction step. Although iron is largely in the Fe(ii) form in the leach solution, the presence of some Fe(iii) could also lower the viscosity of the IL phase, as loading [FeCl_4_]^−^ species in the IL phase gave a reduced viscosity.

**Table 2:** Water content, viscosity and density of the ionic liquid phase from each extraction step in the two-stage cross-current extraction process at 25 °C

Extraction step	Water content (%)	Viscosity (mPa s)	Density (g cm^−3^)
1	6.4 ± 0.6	377 ± 3	0.955
2	5.2 ± 0.5	153 ± 1	0.934

### Precipitation stripping of Fe from the IL phase

To fulfill the circular economy requirement, IL needs to be recycled. To this end, the extracted metal ions (*i.e.*, Fe^2+^ and Fe^3+^) must be stripped from IL phase. Precipitation stripping of iron ions from IL to Fe(OH)_2_ and Fe(OH)_3_ was investigated through the addition of excess aqueous ammonia solution (25 wt%). The precipitation of Fe as Fe(OH)_2_ and Fe(OH)_3_ was successful, reaching 97% precipitation from each of the two phases of the cross-current extraction steps. Due to the high viscosity of the IL system, Fe precipitate may remain in the solution, resulting in lower than 100% precipitation.

To determine the purity and elemental composition of the precipitate, it was dried for 48 h and then digested, followed by ICP-OES analysis. ICP-OES analysis of the precipitate was found to contain no elements other than Fe (33.7 ± 2.7 wt%) and P (0.18 ± 0.01 wt%). The trace amount of P found was due to the IL, as it coated the solid during removal. The precipitate can therefore be assumed as pure Fe(OH)_2_ and Fe(OH)_3_ with no coprecipitation of other metals from the IL. It seems that no iron phosphates have been formed; otherwise a large wt% of P rather than trace quantities would have been measured.

### Reuse of the IL

**Fig. 8:**
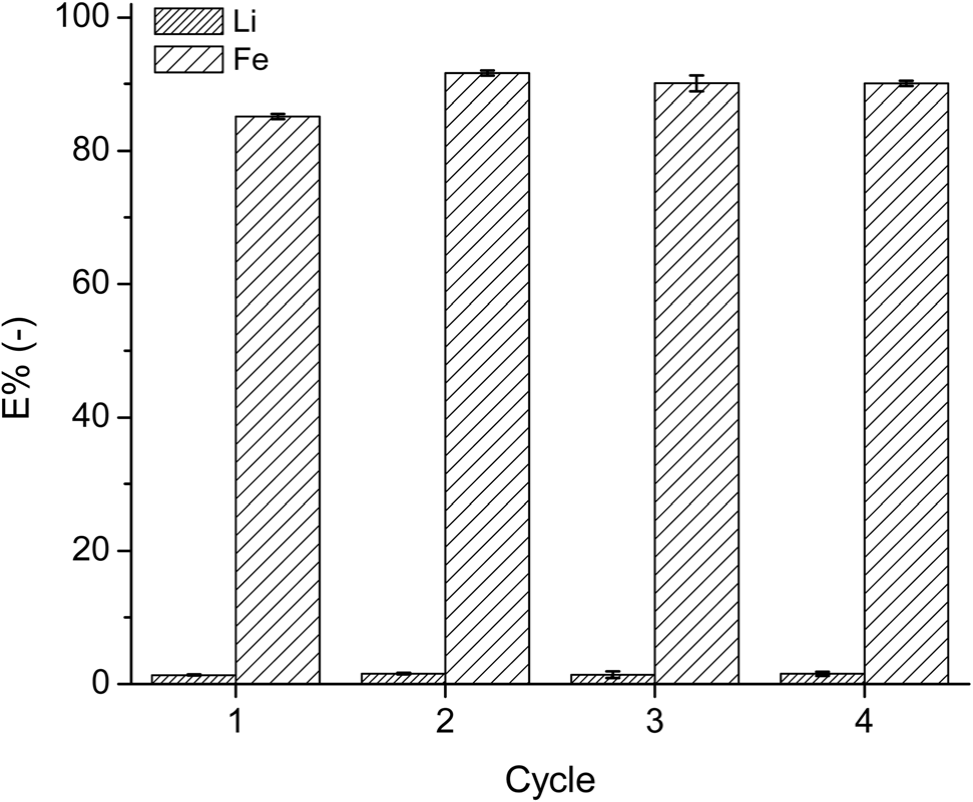
Extraction percentage of Fe and Li by the regenerated IL over 4 cycles, using 4.5 M NaCl leachate at a 3 : 2 aqueous : IL volume ratio, at room temperature.

After successful extraction and precipitation of Fe, the IL was tested for its reusability in the extraction system. An ABS was made using 20 mL of IL and 30 mL of leachate solution and the extraction procedure was the same as the first extraction step in the two-stage cross-current extraction processes. Regeneration of the IL was carried out as previously described. After each extraction step, new leachate solution was added to the regenerated IL. Fig. 8 presents the extraction percentage of Li and Fe by the regenerated IL in each of the 4 cycles.

The efficiency of the IL did not reduce over the four cycles, as in each cycle, the *E*% of Fe remained at approximately 90%. The *E*% of Li remained low at 1%. Moreover, the regeneration procedure and addition of aqueous ammonia solution to the system did not destroy the IL. However, within each step, the IL had to be transferred between multiple reaction vessels, leading to loss of the IL in each step. Therefore, though the viscosity of the IL phase has been reduced significantly compared to the conventional IL extraction system, it is still not low enough to prevent the IL losses during operations.

### Separation of Li from the raffinate solutions

Lithium could be precipitated as lithium carbonate or lithium phosphate through addition of phosphate or carbonate salts (*e.g.*, Na_3_PO_4_ and Na_2_CO_3_).^7,11^ To precipitate as Li_3_PO_4_, control of the solution pH >3 will be required.^38^ Although there should be enough PO_4_^3−^ present in the leachate solution for lithium precipitation, some of them might be lost to the IL phase during the extraction processes. Therefore, the raffinate which was obtained from the 2-stage cross current extraction processes, was first titrated by NaOH (5 M) solution to the designed pH, and then a K_3_PO_4_ solution (2 M) was added for the precipitation of lithium phosphate at 60 °C, as lithium phosphate has a lower solubility at higher temperatures.^38^

**Fig. 9:**
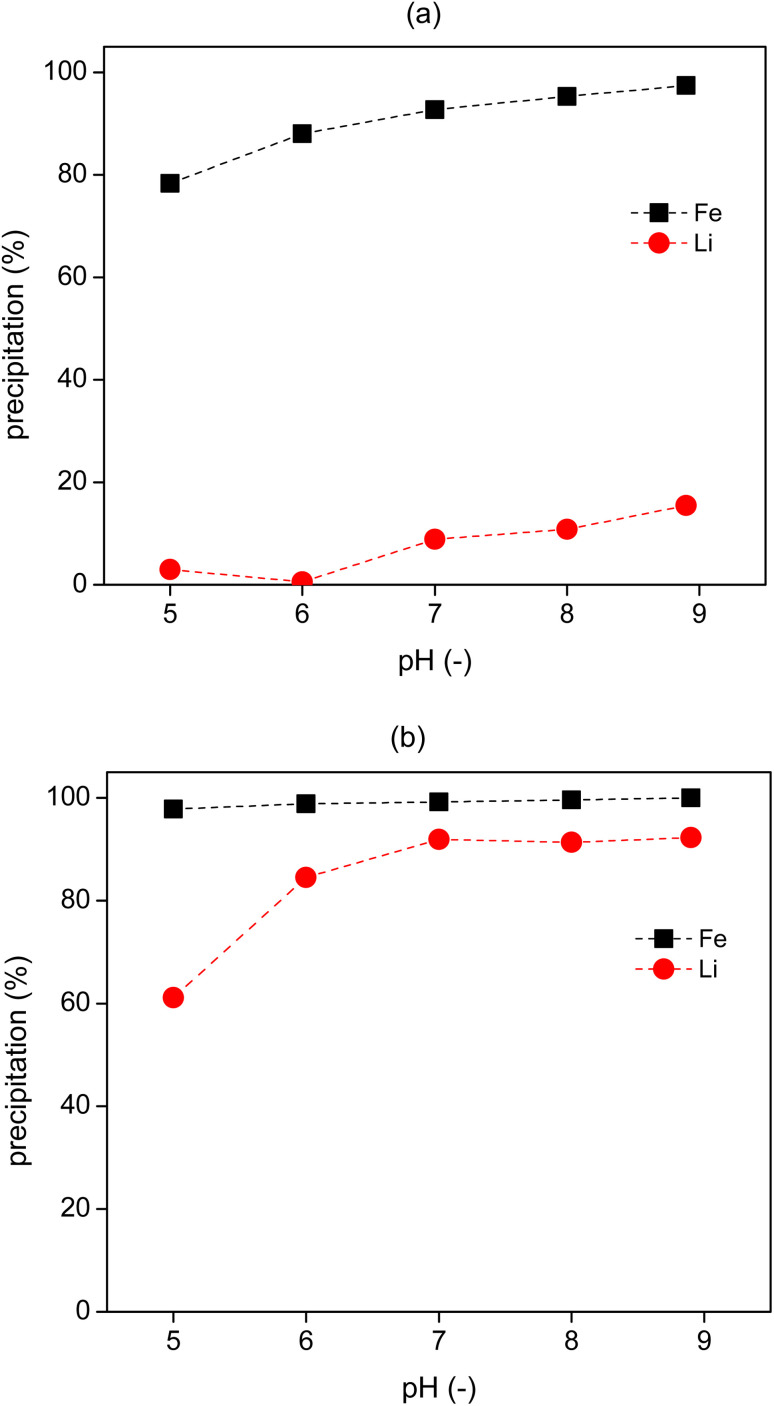
Precipitation of lithium by addition of NaOH at room temperature and thereafter K_3_PO_4_ solution at 60 °C to the raffinate obtained from the 2-stage cross-current solvent extraction process. (a) After adding NaOH to the raffinate, (b) after adding K_3_PO_4_ solution.

Five pH values from 5 to 9 were tested for lithium precipitation. After adding NaOH to the raffinate, precipitates were immediately formed in the solutions, which was mostly Fe precipitate along with some Li precipitate, as confirmed by ICP-OES analysis (Fig. 9). In the following step, about 90% of Li was precipitated by addition of K_3_PO_4_ solution to the pH-controlled solution. By calculating the absolute amount of Li and Fe in the precipitate, it is obtained that the purity of Li in the precipitate was more than 99.5%. A higher pH leads to more lithium precipitation (Fig. 9b), but some of the lithium was lost in the first pH control step, especially at pH 9, where about 15% of lithium was precipitated (Fig. 9a). Therefore, the raffinate could be adjusted to a pH of 6, to allow the precipitation of Fe, then increased to pH 7 for lithium precipitation.

## Conclusions

Overall, it was shown that both Li and Fe in LFP cathode material could be leached by solutions of relatively low HCl concentrations, without any oxidizing agent. The leached iron ions were mostly Fe(ii) with approximately 17% of Fe(iii). An [P_44414_]Cl–NaCl-based ABS can be used to selectively extract iron from leachate solution *via* two extraction steps. Lithium was successfully precipitated from the raffinate as Li_3_PO_4_ with a high purity. Fe could be easily precipitated from the IL phase as a hydroxide precipitate using aqueous ammonia, enabling the regeneration of IL. The regenerated IL could be reused for at least 4 cycles with no decline in extraction efficiency. However, the loss of IL in each cycle was significant. Thus, further study should be conducted to reduce the viscosity of IL, or to develop an intensified process to lessen the loss of IL. Precipitation of Fe from the IL phase by phosphate salts after oxidizing Fe(ii) to Fe(iii) could be further explored, for further reuse of FePO_4_ to prepare new cathode materials.

## Conflicts of interest

There are no conflicts of interest to declare.

## Supplementary Material

RA-014-D4RA00655K-s001
