# Supplementary material for: Recycling of metals from LiFePO4 battery cathode material by using ionic liquid based-aqueous biphasic systems
Source: RSC Adv. 2024 Mar 19;14(13):9262–72. doi: 10.1039/d4ra00655k (PMC10949915; doi:10.1039/d4ra00655k)
Supplement: RA-014-D4RA00655K-s001 [file RA-014-D4RA00655K-s001.pdf]

Electronic Supplementary Material (ESI) for RSC Advances.
This journal is © The Royal Society of Chemistry 2024

# Supplementary information

**Recycling of metals from $LiFePO_4$ battery cathode material by ionic liquid based-aqueous biphasic systems**

Xiaohua Li[†]*, Maia Benstead[‡], Nand Peeters[†] and Koen Binnemans[†]

[†] KU Leuven, Department of Chemistry, Celestijnenlaan 200F, P.O. box 2404, B-3001 Leuven, Belgium.

[‡] Durham University, Department of Chemistry, Durham DH1 3LE, UK

*Corresponding author:

Email: Xiaohua.li@kuleuven.be

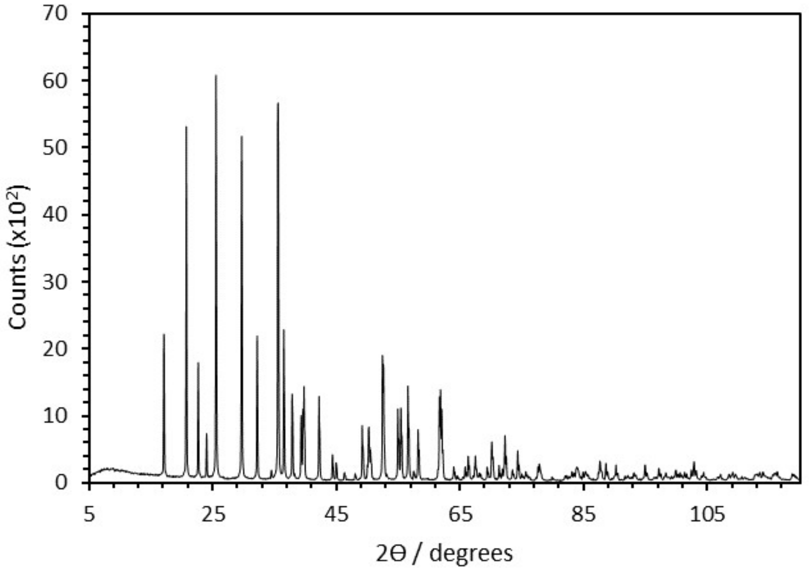


Figure S1: X-ray diffractogram of the raw LFP battery cathode material.

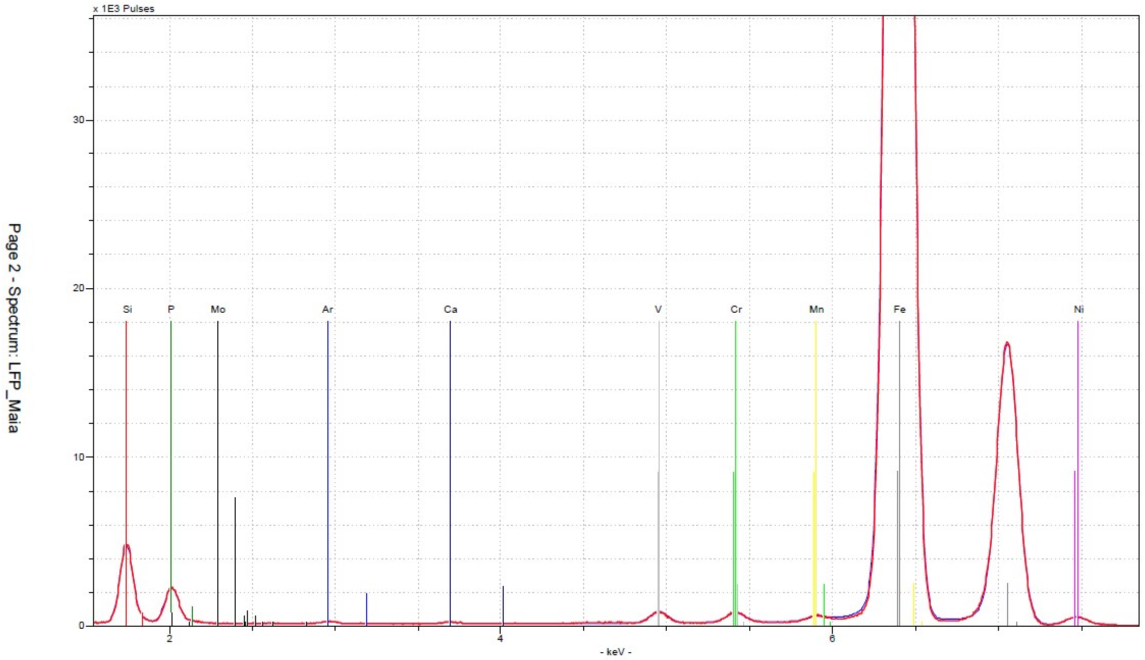


Figure S2. TXRF spectrum of the LFP solid material after suspending in a Triton X-100 solution with Mo source, operating at 50 kV and 600 µA for 300 s.

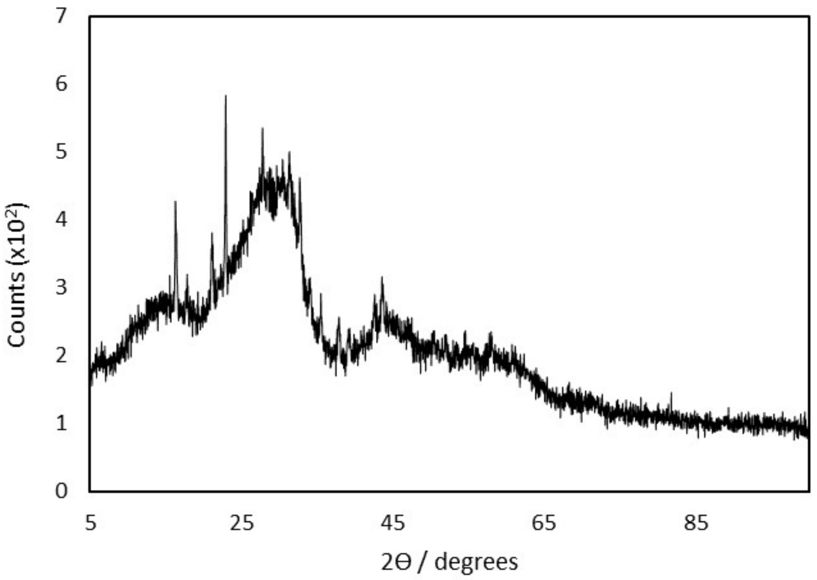


Figure S3: X-ray diffractogram of the leaching residue.

## UV-VIS spectroscopy to determine the oxidation state of iron ions

The iron speciation was studied by UV-VIS analysis spectroscopy by using a colorimetric agent, 1,10-phenanthroline (phen), which can form a colored complex with Fe(II) ions. The experiment was conducted in duplicate. Figure S4 and Figure S5 show the UV-VIS spectra of the standard solutions, the original leachate solutions and the reduced leachate solutions for the duplicate experiments. Both calibration curves used to calculate the concentration values are presented in Figure S6.

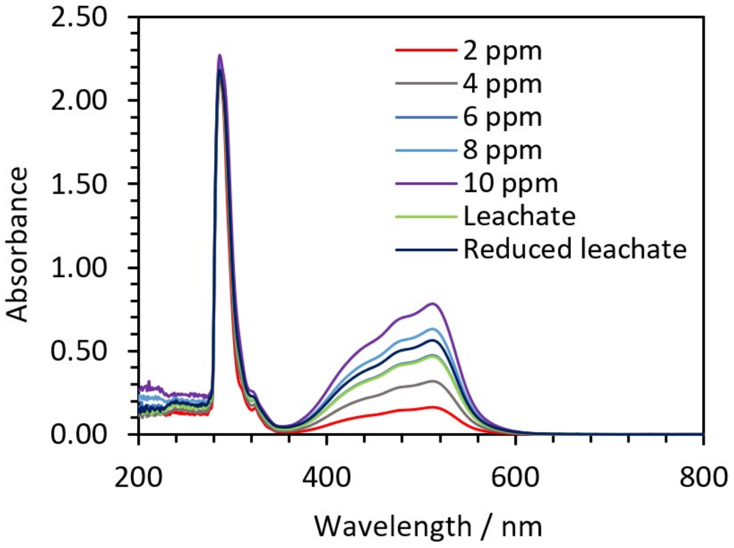


Figure S4. UV-VIS spectra of the Fe(II) standard solutions, leachate and reduced leachate solutions from the 1st experiment.

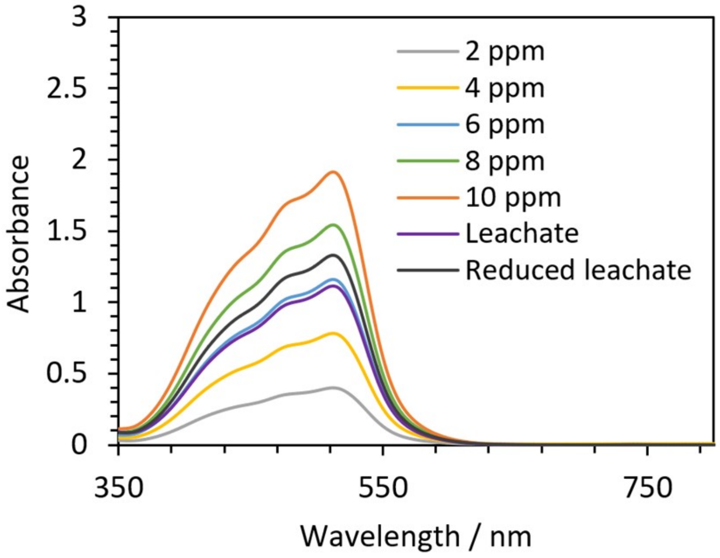


Figure S5. UV-VIS spectra of the Fe(II) standard solutions, leachate and reduced leachate solutions of the 2nd experiment.

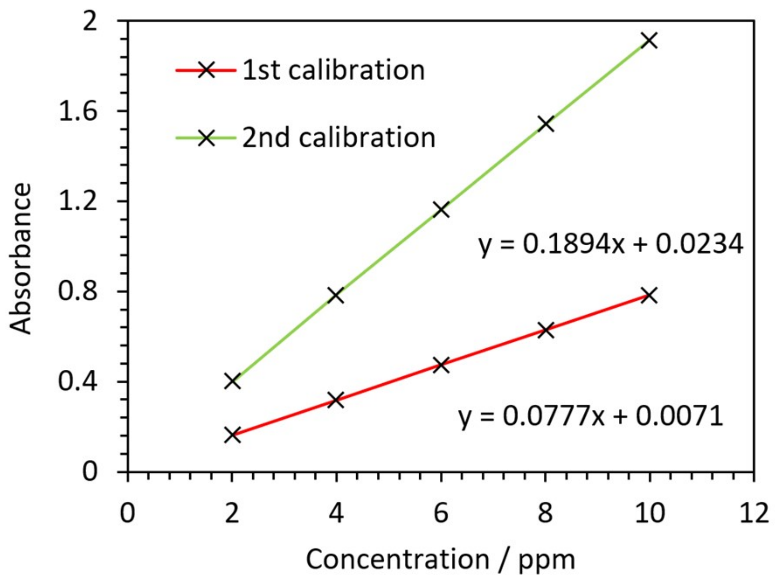


Figure S6. Calibration curve of the absorbance versus concentration of the Fe(II) standard solutions of experiments 1 and 2.

The $[Fe(phen)_3]^{3+}$ complex has a peak absorbance at 512 nm. The absorbance values of the standards at this wavelength were used to construct the calibration curves. In Figure S4, the sharp absorption peak around 300 nm is of the excess 1,10-phenthroline which did not form a complex. Using the calibration curve equations, the concentration of Fe(II) in the unknown samples was determined (Table S1). The duplicate experiments were in agreement within ± 1%. Using the concentration of Fe(II) and Fe(III), it was found that the leachate contained approx. 83 ± 1 % Fe(II) and 17 ± 1% Fe(III).

Table S1: Measured absorbance values for the two leachate samples and the calculated concentration of Fe(II) and Fe(III) for the two repeated experiments.

| | | | UV-Vis concentration (mg/L) | Leachate concentration (g/L) | |
|---|---|---|---|---|---|
| Experiment | Sample | Absorbance | Fe(II) | Fe(II) | Fe(III) |
| 1 | Leachate | 0.466 | 5.91 | 11.81 | 2.53 |
| 1 | Reduced leachate | 0.564 | 7.17 | 14.34 | - |
| 2 | Leachate | 1.114 | 5.76 | 11.52 | 2.29 |
| 2 | Reduced leachate | 1.331 | 6.90 | 13.81 | - |

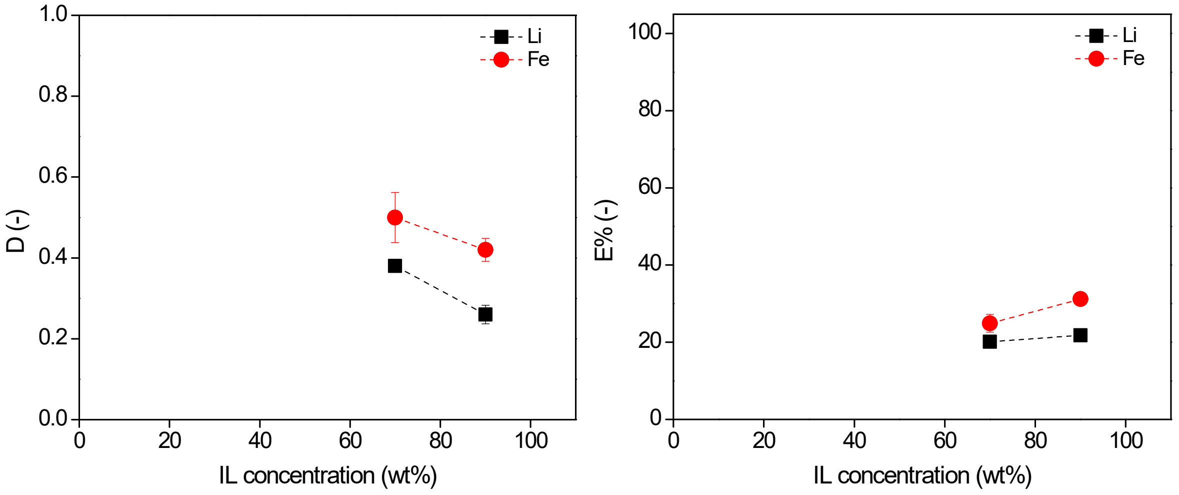


Figure S7. Distribution ratio and extraction percentage of Li and Fe in the $[P_{44414}]Cl–NaCl$ system using leachate with 2M NaCl as a function of the IL concentration, in a 3:2 aqueous : IL volume ratio at room temperature.
